# Progress in genetic mechanisms and precise treatment of neurocutaneous syndrome-related epilepsy

**DOI:** 10.3389/fneur.2025.1642299

**Published:** 2025-09-05

**Authors:** Yang Li, Xiaojie Hu, Xueqing Chen, Yawei Cheng, Yanhong Jiang, Xingchen Wang

**Affiliations:** ^1^The Second Clinical Medical College, Shandong University of Traditional Chinese Medicine, Jinan, China; ^2^Department of Neurology, The Second Affiliated Hospital of Shandong University of Traditional Chinese Medicine, Jinan, China; ^3^Department of Cardiology, Zibo Central Hospital, Zibo, China

**Keywords:** neurocutaneous syndromes, epilepsy, genetic mechanisms, precise treatment, mTOR pathway

## Abstract

Neurocutaneous syndromes are a group of genetic disorders involving the nervous and cutaneous systems, including Tuberous Sclerosis Complex (TSC), neurofibromatosis type 1 (NF1), and Sturge–Weber syndrome (SWS), and others. The incidence of epilepsy, a core clinical manifestation, is significantly higher than in the general population. The purpose of this narrative review is to provide an updated overview of the genetic mechanisms and recent advances in precise treatment for neurocutaneous syndrome-related epilepsy. We conducted a comprehensive search of the PubMed, Scopus, EMBASE, and Web of Science databases using all MeSH terms related to ‘Neurocutaneous Syndromes’, ‘Epilepsy/genetics’, ‘Signal Transduction’, and ‘Precision Medicine’. Selected papers underwent review and risk of bias (RoB) assessment to evaluate core questions. Somatic or germline mutations dysregulate key signaling pathways (e.g., mTOR, Ras-MAPK, PI3K-AKT), inducing malformations of cortical development (MCD) and neuronal-glial dysfunction that collectively form epileptogenic networks. This constitutes the primary pathogenic mechanism underlying neurocutaneous syndrome-related epilepsy. Precise treatment strategies based on molecular mechanisms have achieved breakthroughs: mTOR inhibitors significantly reduce seizure frequency in TSC patients, and cannabidiol (CBD) demonstrates broad-spectrum antiepileptic efficacy in TSC and Dravet syndrome. Advances in surgical techniques, such as multimodal imaging-guided resection, improve outcomes in refractory epilepsy. However, clinical translation faces challenges including technical limitations in detecting mosaic mutations, insufficient specificity of targeted drugs, and interdisciplinary collaboration gaps. Future directions require integrating multi-omics technologies, developing novel gene therapies (e.g., CRISPR-based approaches), and establishing multicenter databases linking genotype–phenotype-treatment responses to advance personalized precision medicine.

## 1 Introduction

Neurocutaneous syndromes (NCS), also termed phakomatoses, represent a heterogeneous group of multisystem genetic disorders characterized by concomitant neurological and cutaneous manifestations. This category encompasses TSC, NF1, SWS, Epidermal Nevus Syndrome (ENS), and neurocutaneous melanosis (NCM), and others (1). These diseases are mostly caused by somatic mutations or germline mutations, resulting in dysregulation of key signaling pathways, which leads to abnormal neural and vascular development, manifesting as MCD, epilepsy, and various skin manifestations (1).

Epilepsy, a hallmark neurological complication of NCS, exhibits strikingly high prevalence across subtypes. In TSC, seizure incidence reaches 80–90%, with approximately 60% of cases progressing to pharmacoresistant epilepsy (2, 3). Similarly elevated rates are observed in SWS (75–90%) and NF1 (4–7%), significantly exceeding population baselines (4, 5). Emerging evidence highlights the pivotal role of somatic mosaicism in cortical malformation-associated epileptogenesis, providing mechanistic insights for targeted interventions (1). Notably, mTOR inhibitors (e.g., sirolimus, everolimus) demonstrate therapeutic efficacy in TSC by modulating aberrant signaling, achieving seizure frequency reduction in 50% of patients and seizure-free outcomes in select cases (6). Concurrently, CBD shows broad antiepileptic potential across refractory epilepsies including TSC, Dravet syndrome, and Lennox–Gastaut syndrome, underscoring the promise of genotype-driven precision therapeutics (7).

Deciphering the genetic architecture and advancing mechanism-based therapies for NCS-related epilepsy hold dual significance: optimizing clinical management through molecular stratification while revolutionizing epilepsy treatment paradigms. Integrating molecular diagnostics with pathway-specific modulation may substantially improve patient prognoses and catalyze the evolution of precision medicine in neurology.

## 2 The genetic mechanisms of neurocutaneous syndrome-related epilepsy

The genetic pathogenesis of neurocutaneous syndromes is primarily attributed to somatic mosaic mutations that dysregulate key signaling pathways.

TSC caused by germline or somatic inactivating mutations in tumor suppressor genes TSC1 (9q34) or TSC2 (16p13.3), manifests through disrupted negative regulation of the mTORC1 pathway. This dysregulation participates in epileptogenesis through multi-level mechanisms: At the neuronal level, mTOR hyperactivation promotes protein synthesis while inhibiting autophagy, leading to neuronal hypertrophy, aberrant dendritic arborization, and synaptic plasticity dysregulation – collectively establishing epileptogenic networks (8, 9). Concurrently, glial dysfunction exacerbates neuronal hyperexcitability through decreased glutamate transporter (GLT-1) expression in astrocytes, causing extracellular glutamate accumulation, and metabolic uncoupling that disrupts the neuron-glial lactate-glutamine cycle (10–13). The TSC1/TSC2 protein complex normally maintains cellular homeostasis, but mutation-induced hyperactivation of mTOR signaling promotes multiorgan hamartoma formation, including cerebral tubers and renal angiomyolipomas (4, 14–17). Furthermore, somatic mosaic mutations in genes such as TSC2 or AKT3 drive PI3K-AKT–mTOR pathway overactivation, inducing focal cortical dysplasia or hemimegalencephaly-critical epileptogenic substrates (18, 19).

NF1 arises from mutations in the NF1 gene (17q11.2), whose product neurofibromin functions as a Ras GTPase-activating protein (GAP) that negatively regulates Ras-MAPK signaling (20, 21). Loss of NF1 activity results in constitutive Ras activation, triggering Schwann cell and glial proliferation. This pathological process disrupts prostaglandin E (PGE) metabolism, elevates neuronal excitability, and creates cortical excitation-inhibition imbalance that underlies spontaneous seizure generation. Characteristic phenotypes encompass café-au-lait macules and skeletal abnormalities (16, 20–22).

**Figure 1:**
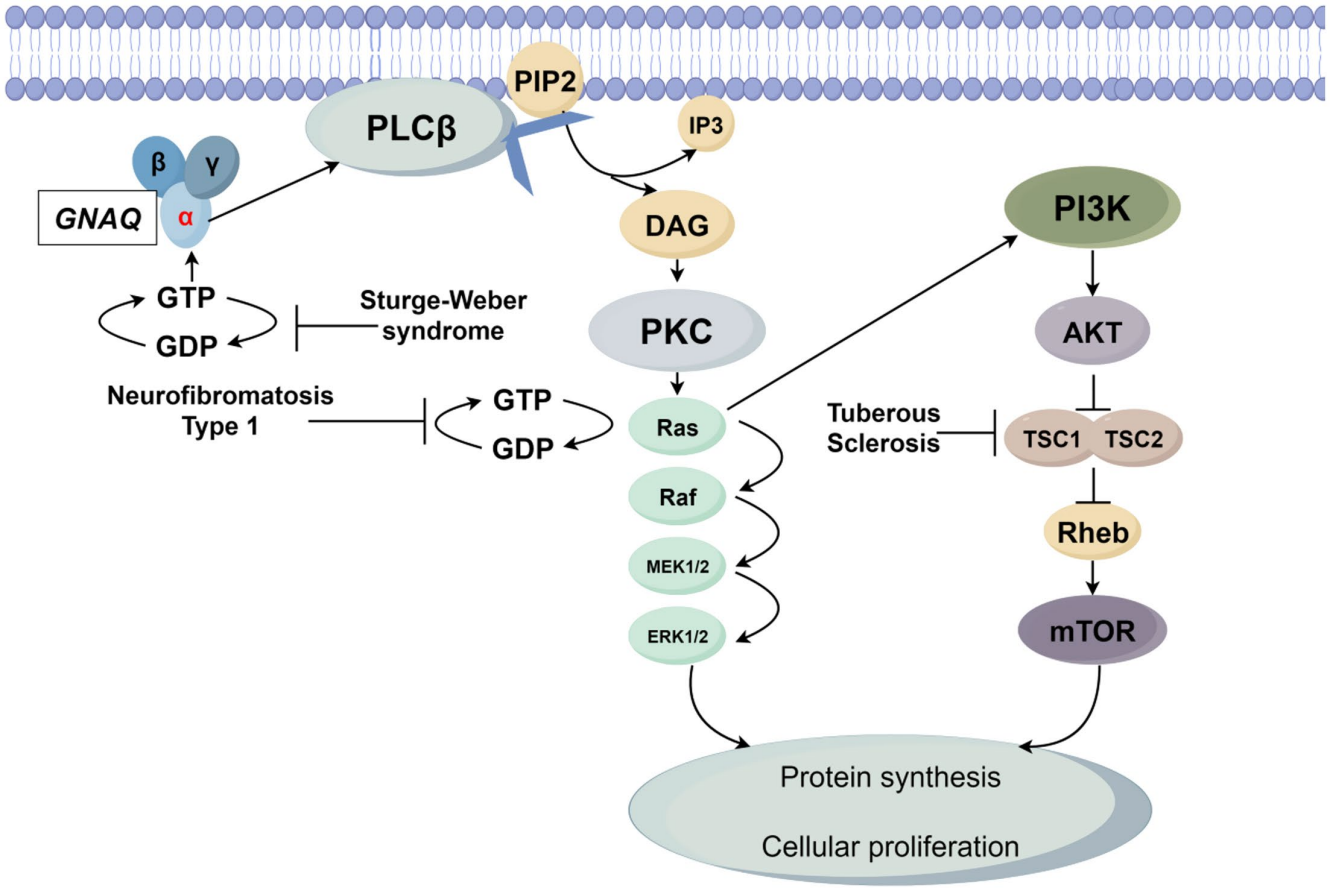
GNAQ is activated upon ligand binding via GDP-GTP exchange, triggering phospholipase Cβ (PLCβ) to hydrolyze membrane-bound phosphatidylinositol 4,5-bisphosphate (PIP2) into diacylglycerol (DAG) and inositol 1,4,5-trisphosphate (IP3). DAG activates protein kinase C (PKC), which in turn promotes GTP binding to the small G protein Ras, rendering it active. Active Ras propagates signals through two key pathways: the mitogen-activated protein kinase (MAPK) cascade (Raf → MEK1/2 → ERK1/2) and the phosphoinositide 3-kinase (PI3K)-AKT–mTOR axis. The MAPK cascade drives ERK1/2 activation, while the PI3K-AKT–mTOR pathway involves AKT-mediated inhibition of the TSC1/TSC2 complex (a Rheb negative regulator), leading to mTOR activation. Ultimately, ERK1/2 and mTOR converge to enhance protein synthesis and drive cell proliferation. Three diseases are linked to network dysregulation: SWS arises from GNAQ gain-of-function mutations, causing persistent GTP binding and constitutive PLCβ pathway activation; NF1 results from NF1 loss-of-function mutations, abrogating neurofibromin (a Ras GTPase-activating protein) and leading to Ras hyperactivation due to impaired GTP hydrolysis; TSC is caused by TSC1/TSC2 mutations, disrupting Rheb regulation and allowing mTOR hyperactivation.

SWS is principally mediated by the somatic mosaic mutation GNAQ c.548G > A (p. R183Q), which activates the Gαq-PLCβ-PKC axis and Rho-ROCK signaling. This molecular derangement induces pathological vascular endothelial proliferation and leptomeningeal angiomatosis, clinically presenting with the classic triad of facial port-wine stains, glaucoma, and neurological calcifications (23–26). Notably, GNAQ mutations exhibit cross-activation of both Ras-MAPK and PI3K-AKT–mTOR pathways, suggesting therapeutic potential for MEK inhibitors (e.g., selumetinib) and mTOR inhibitors (e.g., everolimus) (27, 28). The Figure 1 illustrates the cellular signaling network initiated by the G protein-coupled receptor *α* subunit GNAQ and its molecular association with three genetic diseases, ultimately regulating the process of cell proliferation.

Other neurocutaneous syndromes also exhibit distinct genetic patterns. NCM is associated with somatic mutations in NRAS that dysregulate the MAPK pathway, resulting in aberrant melanocyte proliferation and melanin deposition in cutaneous and leptomeningeal tissues (27, 29, 30). ENS arises from activating mutations in PIK3CA or AKT3, which drive hyperactivation of the PI3K-AKT–mTOR signaling axis, clinically manifesting as epidermal nevi, hemimegalencephaly, and drug-resistant epilepsy (19, 30). Additionally, Cerebrofacial Arteriovenous Metameric Syndrome (CAMS), characterized by metameric arteriovenous malformations, requires differential diagnosis from SWS. Emerging evidence suggests its pathogenesis may involve somatic mutations in vascular patterning genes such as EPHB4 or RASA1 (31).

The shared genetic hallmark of neurocutaneous syndromes lies in somatic mutations disrupting core developmental pathways—including mTOR, Ras-MAPK, and Gαq-PLCβ signaling—leading to pluripotent progenitor cell dysregulation, tissue malformations, and tumorigenesis (32). These mechanistic insights have enabled the successful clinical translation of mTOR inhibitors (rapamycin, everolimus) in TSC-associated epilepsy, demonstrating significant seizure frequency reduction (50% responder rate) and cognitive improvement (33, 34). Emerging therapeutic strategies targeting upstream PI3K-AKT–mTOR pathway components (PIK3CA, AKT1) are undergoing clinical evaluation, heralding new precision medicine approaches (35, 36).

## 3 Progress in precise treatment of neurocutaneous syndrome related epilepsy

Emerging therapeutic strategies targeting mTOR-associated somatic mutations and glial dysfunction have entered translational phases, encompassing both gene-editing technologies (e.g., CRISPR-Cas systems) and pathway-specific inhibitors (37–39). Clinical validation of mTOR inhibitors (e.g., everolimus) in TSC demonstrates substantial therapeutic efficacy, with phase III trials reporting ≥50% seizure frequency reduction in 50% of patients and seizure-free outcomes in subsets, alongside cognitive improvement (37, 40, 41). Notably, expanding applications in RASopathies-related epilepsy (e.g., NF1) are under investigation, though mechanistic validation remains ongoing (40, 42). For GNAQ-mutated SWS, preclinical studies using mTOR inhibitors show reduced neurovascular inflammation in animal models (43), yet clinical evidence remains limited to anecdotal reports (43, 44). Key unanswered questions include: whether GNAQ mutations exert mTOR activation via PI3K-AKT crosstalk (43, 45), and the potential involvement of downstream effectors like HIF-1α in therapeutic responses (45) Future multicenter trials incorporating biomarker-driven designs (e.g., mTOR activation status via phosphor-S6 immunohistochemistry) and combinatorial anti-angiogenic approaches may address current translational challenges (46). Table 1 shows the genetic molecular targets and treatment evidence for various neurocutaneous syndrome-related epilepsy.

**Table 1:** Genetic-molecular targets and treatment evidence for neurocutaneous syndrome-related epilepsy.

Disease name	Pathogenic genes	Core signaling pathways	Targeted drugs	Clinical evidence level	Notes
Tuberous sclerosis (TSC)	TSC1, TSC2	PI3K-AKT–mTOR	Everolimus, Rapamycin	Approved (Phase III Clinical Trial)	Response rate of 50% for TSC-related epilepsy; seizure freedom achieved in some patients (34, 87)
Neurofibromatosis type 1 (NF1)	NF1	Ras-MAPK	MEK Inhibitors (Trametinib)	Clinical Trial Phase (Case Reports)	Effective for NF1-related tumors; epilepsy efficacy mechanism requires validation (21, 78)
Sturge–Weber syndrome (SWS)	GNAQ (somatic mutation)	Gaq-PLCβ-PKC, Ras-MAPK/mTOR	Everolimus, Trametinib	Effective in animal experiments, clinical case reports	Requires verification of whether mTOR inhibition acts through pathways like HIF-1α (5, 52)
Epidermal nevus syndrome (ENS)	PIK3CA, AKT3	PI3K-AKT–mTOR	mTOR Inhibitors (Everolimus)	Preclinical Studies	Mechanistic correlation requires further validation (37, 42)
Neurocutaneous melanosis (NCM)	NRAS (somatic mutation)	MAPK	MEK Inhibitors (Trametinib)	Theoretical support, no clinical data	Correlation between melanocyte proliferation and epilepsy remains unclear (28–30)
Focal cortical dysplasia (FCD II)	MTOR, DEPDC5	PI3K-AKT–mTOR	Everolimus (Experimental Use)	Preclinical Studies	mTOR pathway mutations present in 60% of patients; targeted therapy still in exploratory stage (45, 88, 89)
Hemimegalencephaly (HME)	AKT3, PIK3CA	PI3K-AKT–mTOR	mTOR Inhibitors	Effective in animal models	Fetal somatic mutations lead to abnormal pathway activation; requires optimized dosing regimens (36)
Cardiofaciocutaneous syndrome (CFCS)	BRAF, MAP2K1	Ras-MAPK	MEK Inhibitors (Trametinib)	Clinical Trial (Reduced seizure frequency)	neurodevelopmental impact; long-term safety to be verified (73, 90)

Cutting-edge methodologies integrating single-cell transcriptomics and spatial proteomics are revolutionizing our understanding of epileptogenic niches. These techniques enable high-resolution mapping of neuron–glia-vascular unit interactions within seizure foci, identifying novel therapeutic targets such as senescent cell populations and dysregulated lactate shuttling (9, 47, 48). This paradigm shift from histomorphological characterization to molecular network analysis provides critical insights for refractory epilepsy management. Ultimately, the convergence of multi-omics profiling, advanced neuroimaging, and clinical phenotyping will catalyze the development of personalized therapeutic frameworks for neurocutaneous syndrome-related epilepsy.

In the domain of novel antiepileptic therapeutics, CBD has emerged as a pharmacological intervention with multi-target mechanisms, including modulation of AMPA, GABA, and GPR55 receptors, demonstrating significant therapeutic potential (41, 49, 50). CBD antagonizes G protein-coupled receptor 55 (GPR55), inhibiting its mediation of intracellular calcium release and mTOR pathway activation. This reduces downstream protein synthesis, regulates autophagy processes, clears abnormal protein accumulation, and alleviates abnormal neural proliferation and seizures (51). Currently approved for Dravet syndrome, Lennox–Gastaut syndrome, and TSC-associated epilepsy, CBD adjunctive therapy achieves ≥50% seizure reduction in 50–60% of patients, with particularly notable efficacy in controlling epileptic spasms among TSC patients (45–50% responder rate) (41, 52, 53). However, hepatotoxicity risk requires vigilant monitoring when co-administered with valproic acid, evidenced by elevated liver enzyme levels (54–56). While short-term safety profiles appear favorable, long-term administration necessitates individualized risk–benefit assessment, particularly regarding cardiovascular parameters and pharmacokinetic interactions. The current evidence base lacks extended longitudinal data beyond 5-year follow-up, underscoring the need for syndrome-specific outcome studies (57). Notably, advancements in precision dosing technologies, including ultra-performance liquid chromatography–tandem mass spectrometry (UPLC-MS/MS), are revolutionizing personalized therapeutic regimens (58).

Surgical innovations have substantially enhanced therapeutic outcomes through multimodal localization strategies. The integration of 18F-fluorodeoxyglucose positron emission tomography (18F-FDG PET; sensitivity 70–80%) with magnetoencephalography (MEG) enables precise epileptogenic zone delineation, particularly for TSC cortical tubers (44). Resective surgery achieves seizure-free outcomes in 60–70% of unifocal TSC cases, while hemispheric disconnection procedures yield 80% seizure freedom rates in SWS, albeit with potential neurological sequelae requiring careful preoperative evaluation (43, 44). Emerging evidence suggests that mTOR pathway activation status, as determined by immunohistochemical markers, may serve as a predictive biomarker for postoperative recurrence (37).

The optimization of individualized therapeutic regimens faces dual challenges in bridging mechanistic research and clinical translation. Current investigations into epileptogenic mechanisms have yet to fully elucidate critical pathway interactions. For instance, the causal relationship between BRAF/MAP2K1 variants and epileptic encephalopathy in CFCS remains ambiguous, significantly impeding targeted drug selection (40, 44, 59). Existing therapeutic strategies remain fragmented, with most antiseizure medications (ASMs) primarily addressing symptomatic management rather than correcting underlying genetic defects (e.g., avoidance of sodium channel blockers in SCN1A mutations), while advanced interventions such as gene replacement therapy remain confined to preclinical development (60, 61). This therapeutic impasse is further compounded by the lack of standardized efficacy evaluation systems, particularly regarding dynamic monitoring of biomarkers such as electroencephalographic (EEG) signatures and molecular imaging parameters (62, 63). Establishing multimodal “genotype–phenotype-treatment response” databases, advancing molecular stratification-based clinical trials (e.g., MEK inhibitors for RASopathies-associated epilepsy), and developing companion diagnostic tools emerge as pivotal strategies to overcome these barriers (40, 64).

The development of novel targeted therapies demonstrates diversification but confronts technical and commercial complexities. RAS-MAPK pathway inhibitors (e.g., selumetinib) exhibit seizure frequency reduction potential in CFCS-related epilepsy, though their long-term neurodevelopmental impacts and safety profiles require rigorous validation (40, 65). As emerging experimental strategies CRISPR-based gene editing technologies hold transformative potential for neurocutaneous syndromes. Precision manipulation of disease-associated genes (e.g., TSC1/TSC2, NF1) enables accurate modeling of patient-specific mutations and elucidation of epileptogenic pathways (66). Preclinical studies confirm that CRISPR-Cas9-mediated correction of TSC1/TSC2 mutations effectively suppresses mTOR pathway hyperactivation and reduces seizure incidence, providing mechanistic validation for gene therapy (57, 67). However, ethical concerns persist across developmental stages, including risks of off-target genomic alterations (e.g., unintended CRISPR/Cas9 activity), immune responses to viral vectors (e.g., AAVs), and unpredictable neurocircuitry remodeling (68–71). Technical hurdles further include achieving stable regulation of vector-mediated gene expression (72) and maintaining excitatory/inhibitory balance during neural network modulation (71). While AAV-based strategies targeting SCN1A and MECP2 mutations face challenges in blood–brain barrier penetration and immunogenicity (73, 74), mTOR inhibitors like everolimus demonstrate dual antiepileptic and antitumor efficacy in tuberous sclerosis, though optimal dosing regimens require refinement (29, 75). Notably, most therapies remain mutation-specific with limited capacity to reverse established neural circuit abnormalities, compounded by recruitment challenges and limited profitability in rare disease drug development (60, 64).

Implementing multidisciplinary care systems is paramount for optimizing therapeutic outcomes. Neurocutaneous syndromes’ multisystem involvement (neurological, dermatological, ocular) demands coordinated expertise, yet current collaboration models exhibit critical deficiencies. Diagnostic delays persist in conditions like Aicardi syndrome due to underrecognized dermal/retinal manifestations (45, 76), while discrepancies between neurologists’ genetic counseling proficiency and geneticists’ epilepsy expertise result in fragmented decision-making (29, 77). Data silos across genomic, imaging, and histopathological platforms further obstruct comprehensive evaluations (62, 78). Addressing these systemic gaps requires establishing specialized neurocutaneous syndrome centers with standardized multidisciplinary workflows (e.g., tumor boards for TSC), integrated clinical databases, and genetics competency training programs to cultivate an integrated diagnostic-therapeutic ecosystem (62, 76).

## 4 Discussion

Research on genetic mechanisms of neurocutaneous syndrome-related epilepsy has elucidated pathogenic pathways across distinct syndromes. TSC1/TSC2 mutations in TSC activate mTOR signaling to promote epileptogenesis, establishing molecular foundations for targeted therapies (79). Similarly, the CFCS, driven by RAS-MAPK signaling pathway variants (BRAF, KRAS), demonstrates epileptogenic mechanisms involving neuronal hyperexcitability and synaptic plasticity dysregulation (40, 65). The pathogenic mechanisms of different syndromes are both specific (such as over-activation of the mTOR pathway in TSC and cross-activation of multiple pathways by GNAQ mutations in SWS) and common (such as cross-disease effects of glial cell metabolism imbalance and excitotoxicity). Their interactions form a complex network in epileptogenesis. These advances not only delineate genotype–phenotype correlations [e.g., specific mutations associated with Infantile epileptic spasms syndrome (IESS) versus focal epilepsy (80, 81)], but also directly inform therapeutic development. MEK inhibitors like selumetinib targeting the RAS-MAPK pathway exhibit clinically significant seizure reduction in CFCS patients (40). Genetic diagnostics further optimize antiepileptic drug selection, exemplified by avoiding sodium channel blockers in SCN1A mutation carriers while prioritizing valproate (77, 82).

Clinically, synergistic approaches combining CRISPR-based gene editing, single-cell sequencing, and molecularly targeted therapies (mTOR inhibitors, ion channel modulators) show transformative potential (59, 61, 73). Establishing dynamic genotype–phenotype-treatment response databases will refine personalized regimens (80, 83), while prospective trials must validate the long-term efficacy and safety of emerging inter ventions like neuromodulation and gene replacement therapies (83, 84). Future directions should address genetic-psychiatric comorbidities (autism spectrum disorders, cognitive impairment) through integrated treatment paradigms that simultaneously optimize seizure control and neurodevelopmental outcomes (85, 86).

## 5 Conclusion

This review systematically clarifies the core pathogenic mechanisms and precise treatment strategies of neurocutaneous syndrome-related epilepsy, and reveals a molecular pathological network characterized by abnormal activation of mTOR, Ras-MAPK and other signaling pathways. Somatic/germline mutations drive the formation of epileptogenic networks by regulating neuron-glial dysfunction and cortical development malformations; interventions targeting pathways such as mTOR and MEK have demonstrated clinical potential. Current research faces challenges such as insufficient accuracy in the detection of chimeric mutations, unclear neurodevelopmental effects of targeted drugs, and lack of efficacy evaluation systems, which limit the in-depth implementation of personalized treatment.

Analyze the spatio-temporal specific activation patterns of signal pathways in the epileptogenic microenvironment, identify the core regulatory nodes of abnormal neuron-glial metabolic coupling; develop precise hierarchical treatment plans based on mutation lineages, combining CRISPR gene editing and multimodal imaging technology to achieve etiological intervention; Build a dynamic database of “genotype-treatment response” and verify the long-term safety and neuroprotective effects of new therapies (such as MEK inhibitors and gene replacement therapy) through multi-center collaboration. These studies will promote the transformation of diagnosis and treatment models from symptom control to pathological mechanism targeting, laying an important foundation for the precise treatment practice of neurocutaneous syndrome-related epilepsy.
